# Beyond image quality: Patient experience with long-axial field-of-view and standard PET/CT systems

**DOI:** 10.1007/s00259-026-07873-8

**Published:** 2026-04-11

**Authors:** Daniel Sauerbrunn, Norbert Schäffeler, Julia Sekler, Johann Jacoby, Konstantin Nikolaou, Christian la Fougère, Brigitte Gückel

**Affiliations:** 1 Department of Diagnostic and Interventional Radiology, University Hospital Tübingen, 72076 Tübingen, Germany; 2 Psycho-Oncology Section, Department of Psychosomatic Medicine and Psychotherapy, University Hospital Tübingen, 72076 Tübingen, Germany; 3 Institute for Clinical Epidemiology and Applied Biometry, Faculty of Medicine, Eberhard Karls University Tübingen, 72076 Tübingen, Germany; 4 Comprehensive Cancer Center (CCC-TS), University Hospital Tübingen, 72076 Tübingen, Germany; 5 Cluster of Excellence iFIT (EXC 2180) “Image Guided and Functionally Instructed Tumor Therapies”, University of Tübingen, 72076 Tübingen, Germany; 6 German Cancer Research Center, German Cancer Consortium DKTK, Partner Site Tübingen, Tübingen, Germany; 7 Department of Nuclear Medicine and Clinical Molecular Imaging, University Hospital Tübingen, 72076 Tübingen, Germany; 8 Diagnostic and Interventional Radiology, University Department of Radiology, Hoppe-Seyler-Str.3, 72076 Tübingen, Germany

**Keywords:** PET/CT, Long axial field of view (LAFOV), Subjective perception, Visual analogue scale, Patient survey

## Abstract

**Purpose:**

This study examined how patients subjectively perceive and experience PET/CT scans, as well as the impact of psychological stress, demographics, and the use of two different scanner types (a standard field-of-view (SAFOV) scanner (Biograph mCT) and a new-generation PET/CT scanner with a long axial field-of-view (LAFOV) (Biograph Vision Quadra)).

**Methods:**

This prospective clinical trial involved asking 1,003 patients to complete questionnaires about their perceptions and potential side effects following a PET/CT scan by using visual analogue scales (VAS). Patients were also asked to complete standardized questionnaires on stress, anxiety and depression. 620 of the total number of 1,003 patients were scanned with the SAFOV scanner and 383 with the LAFOV scanner. 84 patients who had multiple scans experienced both scanners Logistic regression models were applied to determine predictors on patients’ ratings of their scan experience.

**Results:**

Several factors negatively influenced various aspects of the scan experience: The most important of these were older age, male gender and subjective feelings of tension during the scan. Clinically elevated depression scores and older age can predict patients feeling generally not well cared for. Patients general stress and anxiety tend to play a subordinate role. Acute tension experienced during the scan itself has a far greater influence; particularly on tolerance of the scan time and positioning, and can even worsen the assessment of the appropriateness of the equipment. The LAFOV scanner was found to be superior to the conventional SAFOV scanner in terms of patient evaluations, primarily due to its ability to performe scans more quickly.

**Conclusions:**

From the patient’s perspective, scan time is the most important variable that can be adjusted to improve the PET/CT examination experience. The use of LAFOV scanners makes it possible to significantly reduce the scan time for most standard PET/CT examinations, thereby greatly improving patient comfort. Furthermore, it would be important to take measures to alleviate the patient’s acute tension during the examination.

**Trial registration number: German clinical trials register:**

DRKS00026163 (23-Aug-2021).

## Introduction

PET/CT imaging plays an increasingly important role, particularly in oncology; where indications include primary diagnosis, staging and therapy follow-up. Based on PET/CT, individual therapy strategies are determined that can mark relevant life milestones for the patient. The expectation of a potentially bad diagnosis, but also the examination process itself, can cause anxiety and stress. Therefore, a pleasant examination atmosphere and a high level of comfort during the examination itself are extremely important in order to allay the patient’s fears, encourage cooperation during the examination and, ultimately, achieve optimal image quality.

Comfort is regularly highlighted in most reviews of PET/CT scanners, but there are only a few systematic surveys of patients about their experience of the scan [1]. Although the methods of these studies were different and difficult to compare, they all agree that patients may experience anxiety before, during or after a PET/CT scan. Self-reports of sensory adverse events are rare and mainly available only from high-field MRI studies, most of which were conducted in healthy volunteers [2–7]. In one of our own studies, about 10% of patients reported one of the following discomforts during PET/CT: claustrophobia, headache, nausea, vertigo, ear noises or heat sensations [8]. Looking at the correlations between patient-dependent parameters and patient ratings, no effects of age, sex, BMI, or actual scan times on the results were observed, except for one: male patients had a lower tolerance for their subjectively experienced scan times than female patients [8]. However, there appear to be a number of factors that may influence anxiety associated with PET/CT scans. These include the clinical situation in which patients find themselves (especially staging) [1], worry about the results of the scan [9, 10], whether it is the first PET/CT scan or repeated scans [11], fear of certain procedures (e.g. radiation, lying in a narrow “tube”, positioning, scan duration and waiting times) [8–10, 12] andthe gender of the patient. Although large surveys of cancer patients have shown that female patients generally experience higher levels of anxiety [13–16], some studies of PET/CT scans have shown that here men tend to be more anxious [1].

Our study used standardised questionnaires to ask about psychological distress in a group of mainly cancer patients. In addition, patients were specifically asked about their experience of the PET/CT scan and some side effects they may have experienced. For reasons of feasibility and statistical robustness, visual analogue scales (VAS) were used to quantify these patient scores.

We also compared two different scanner models in terms of patient feedback. The new-generation PET/CT scanner with a long axial field-of-view (LAFOV) offers technical advantages over a SAFOV PET/CT scanner due to its significantly higher sensitivity (up to 10 times greater) [17, 18]. This enables the adjustment of imaging parameters by reducing the injected activity and/or shortening the acquisition time [19]. The latter in particular could have a significant impact on patient comfort.

The main aims of this study were to answer or test the following questions and hypotheses:

- What PET/CT-related side effects and discomfort do patients report, and are these influenced by demographic factors or psychological distress?
- Do patients rate conventional SAFOV PET/CT scanners and the recently introduced LAFOV PET/CT scanners differently in terms of comfort?
- Is it possible to pre-identify patients who need more intensive support and care before and during PET/CT scans?

## Materials and methods

### Study population

A total of 1,003 adult patients who underwent SAFOV PET/CT examinations at the Department of Radiology in Tübingen, Germany, completed questionnaires about their subjective perceptions and sensory side effects. Examinations were performed using either a Biograph mCT (Siemens Healthcare, Erlangen, Germany) between November 2021 and March 2022 (*n* = 620) or a Biograph Vision Quadra (Siemens Healthineers) between January and June 2023 (*n* = 383). Of these patients, 84 experienced both scanners as they underwent multiple scans.

Patients were also asked to complete standardized questionnaires about their feelings of anxiety, depression and distress. The clinical trial was approved by the Institutional Ethical Review Board at the University of Tübingen, Germany (N° 504/2021BO2). Written informed consent was obtained from all subjects before they participated in the study. They were mainly patients with oncological diseases with a median time of 22 months since the first diagnosis. Due to the high prevalence of prostate cancer patients (29%), more men (69%) than women (31%) participated in this study. The next two largest patient groups were those with melanoma (14%) or neuroendocrine tumours (NET) (13%). 90% of the patients had only one PET/CT scan during the study period; but as this was also a longitudinal study 10% of the patients had several PET/CT scans. The questionnaires were to be completed at each PET/CT examination. Patient characteristics are summarized in Table 1.

**Table 1:** Patient characteristics

Variables	Total study sample (*n*=1,003)	Women (*n*=306, 31%)	Men (*n*=697, 69%)
Mean age in years	63	59	64
(SD, range)	(13.6, 18-93)	(14.1, 19-84)	(13.0, 18-93)
Non-cancer patients (*n*, %)	16 (1.6%)	8 (2.6%)	8 (1.1%)
Unknown Diagnosis (*n*, %)	15 (1.5%)	7 (2.3%)	8 (1.1%)
Cancer patients (*n*, %)	972 (96,9%)	291 (95.1%)	681 (97.7%)
of that			
Prostate carcinoma	292 (29.1%)	-	292 (41.9%)
Melanoma	143 (14.3%)	68 (22.2%)	75 (10.8%)
Neuroendocrine tumors	130 (13%)	57 (18.6%)	73 (10.5%)
Non-Hodgkin lymphoma	93 (9.3%)	41 (13.4%)	52 (7.5%)
Hodgkin lymphoma	30 (3%)	19 (6.2%)	11 (1.6%)
Others	284 (28.3%)	106 (34.6%)	178 (25.5%)
PET/CT examinations (n)^1^	1,115	355 (32%)	760 (68%)
Single examination 1x	908 (90%)	267 (87%)	641 (92%)
Multiple examinations 2x	79 (8%)	30 (10%)	49 (7%)
3x	16 (2%)	9 (3%)	7 (1%)
number of patients per scanner			
SAFOV only	536 (53%)	166 (54%)	370 (53%)
LAVOV only	383 (38%)	114 (37%)	269 (39%)
SAFOV & LAFOV PET/CT^2^	84 (8%)	34 (11%)	50 (7%)

^1^During the course of the study, repeated PET/CT scans were performed on some patients. The questionnaires were completed at each PET/CT examination. ^2^In this subcohort, patients received a PET/CT scan on the SAFOV scanner and the next follow-up examination on the LAFOV scanner

### PET/CT

PET/CT scans were performed according to the appropriate clinical indication using a SAFOV PET scanner (Biograph mCT) with a PET field of view of 21.8 cm, a tunnel length of 136 cm and a bore size of 78 cm [20]. The LAFOV PET scanner (Biograph Vision Quadra) was used in addition to the SAFOV scanner in a subcohort of patients who underwent multiple scans (*n* = 84, Table 1) following Biograph mCT replacement. This allowed us to directly compare the two devices in terms of patient feedback. The LAFOV PET scanner has the same bore size but a different axial field of view (106 cm) and tunnel length (235 cm) and allows a shorter average scan time of approx. 8 min.

Diagnostic contrast-enhanced CT or low-dose unenhanced CT scans were performed, depending on the clinical indication and previous imaging. Patients fasted for at least 6 h before receiving an intravenous injection of [^18^F]FDG. Other tracers used were mainly [^18^F]PSMA-1007, [^18^F]SIFA-TATE, according to current guidelines.

The standard protocols for [^18^F]FDG and [^18^F]PSMA-1007 for each scanner are as follows: CT examination on a SAFOV scanner was performed as whole-body or partial-body scan depending on clinical issue. PET was acquired with 3 MBq/kg over 6–8 bed positions (2.5–3.0 min/bed), totaling an average scan time of 15–25 min. CT examination on a LAFOV scanner was performed as whole-body scan (base of the skull to the upper thigh). PET was acquired with 2 MBq/kg over 1–2 bed positions (5 min/bed) totaling an average scan time of 5–10 min. The preparation time for patients is the same for both scanners used (i.e. establishing i.v. access, applying tracer and uptake time, positioning the patient on the scanner table).

### Questionnaires for self-reporting of side effects and perceptions during the PET/CT scan

Patients were asked to complete questionnaires about their examination experience, anxiety and depression. Part I assessed examination-related perceptions by asking the following questions: “Was the information given to you by your doctor comprehensive and coherent?”, “Did you feel well looked after during the examination?”, “Were you free from anxiety during the examination?”, “Was the positioning comfortable for you?”, “Was the duration of the examination bearable?”, “Was the total time spent on the examination OK?”, “Are you convinced that the examinations performed with this scanner are state of the art?”, and “Do you find the equipment in the examination rooms and scanner suitable for the most modern medical diagnostics?”. Part II covered seven possible sensory side effects, namely the occurrence of claustrophobia, tension, headaches, vertigo, nausea, pain and urinary urgency. For self-report, VAS were used and presented as 100 mm (horizontal) lines anchored by two verbal descriptors: “completely false” (negate statement) and “completely true” (confirm statement) and patients were asked to indicate their rating at a point between 0 and 100 mm at their discretion. For clarity, the anchors were also marked with icons showing “” or a “”, respectively. Scores were measured from the zero anchors to the patient’s mark. Due to their multiple discrimination levels, VAS proved to be a useful and sensitive tool for assessing subjective perception and sensations experienced during imaging examinations [8]. Patients completed the self-report questionnaires Part I and Part II immediately after the PET/CT scan. These parts of the questionnaire were previously developed by the authors for a study comparing patients’ experiences with PET/CT and PET/MR examinations [8]. Here, however, a pre-test was included and the questionnaire was administered to 138 patients to rule out potential problems or incomprehension. Based on the feedback, a short instruction with illustrative examples for the completion of the VAS was provided. The median VAS scores were defined as a cut-off values to distinguish between patients with and without side effects or discomfort.

### Questionnaires to record anxiety, depression and stress

Part III of the questionnaire contained a short form of the German version of the standardized Patient Health Questionnaire (PHQ-4) with two questions each on depression (PHQ-2) and anxiety (Generalized Anxiety Disorder (GAD)-2) [17]. These questionnaires have been validated and can be used with both healthy adults and patients in different settings to identify clinically relevant levels of anxiety, stress or depression [21, 22]. Patients were asked to consider the period of the last two weeks when answering the questions. The GAD-2 sub-score was calculated by adding the scores of the two questions into “Little interest or enjoyment in your activities” and “Dejection, melancholy or hopelessness”. Response options reflect how often an item applied to them on a 4-point scale: “not at all” (score: 0), “on some days” (score: 1), “on more than half of the days” (score: 2) or “almost every day” (score: 3). The GAD-2 score therefore ranges from 0 to 6. The PHQ-2 score was calculated similarly from questions about “nervousness, anxiety or tension” and “not being able to stop or control worrying”. The sum of GAD-2 and PHQ-2 gives the PHQ-4 score (range 0–12). For the PHQ-2 and the GAD-2, scale scores of ≥ 3 were used as cut-off points between the normal range and probable cases of depression or anxiety, respectively [21, 23, 24].

In addition, the German version of the standardized Perceived Stress Questionnaire (PSQ-20) was used in Part IV. It contains 20 questions, including five questions on each of the following emotions: worry, tension, joy and demands. Scale 1 (Worry) covers worry, anxiety about the future, and feelings of despair and frustration. Scale 2 (Tension) explores tense restlessness, exhaustion, and the lack of relaxation. Scale 3 (Joy) deals with positive feelings of challenge, joy, energy, and security. Scale 4 (Demands) covers perceived environmental demands, such as lack of time, pressure, and overload. Patients should consider how often an item has applied to them in the last four weeks. If at least three out of five responses were missing or unusable, the corresponding (sub)score was not generated. In instances where a minimum of three out of five questions had been answered, the missing answers were supplemented by the mean value of the answers provided by the patient. In 199 cases imputation was necessary. Scores were calculated in REDCap [25, 26]. All 20 questions can be combined to give an overall stress score, as well as individual scores for each of the emotions mentioned [18]. As the stressors are generic factors, the questionnaire can be administered to different clinical and healthy adult samples in different settings. For the PHQ-20, scale scores have been suggested as cut-off points between the normal range and clinically relevant cases of distress [22].

Patients filled out Parts III and IV of the questionnaire while waiting for their PET/CT scan. These refer to a period of up to four weeks prior to the completion date (e.g. Part IV asks, “How often has this statement applied to your life in the last 4 weeks?”).

### Data collection

All patient-related data such as sociodemographic and morphometric information (age, sex, indication for PET/CT, diagnosis, relevant medical data, BMI etc.) were collected from in-house clinical reports. The study patients’ data were exported from the hospital’s primary radiology information system (RIS) in pseudonymized form in order to determine, for example, the age and sex distribution in the overall population. The questionnaires were paper-based and collected prospectively and consecutively. All questionnaires used remained the same throughout the study. Study data were collected and managed by qualified and approved personnel according to predefined standard operating procedures (SOP) using REDCap electronic data capture tools hosted at Yale University [25, 26]. The accuracy of the data entry was checked by double data entry (DDE).

### Statistics

Unless otherwise stated, patients’ questionnaires and images from their first PET/CT scan within the study period were used for all evaluations and correlation analyses.

To determine possible impact factors (predictors) on patients’ ratings of their scan experience logistic regression models have been applied. The median patient rating of each question was set as a cut-off value to transform the items into dichotomous data. Patient ratings equal to the median were counted to the “positive” outcome group (e.g. no claustrophobia). Cook´s distance was calculated to check for datapoints with large residuals (outliers: Cook´s distance > 1). Multicollinearity was tested by calculating correlation coefficients (Spearman’s correlation) between the predictors and checking if those exceeded *r* > 0.8. Wald test was applied to calculate the probability of error for significance of predictors (*p* < 0.05 was considered significant).

For those patients who underwent consecutive questioning on both scanners (SAFOV and LAFOV PET scanner) comparison of scan experience ratings was performed by Wilcoxon signed rank test. This method was selected after viewing the distribution of the “rating differences” between SAFOV and LAFOV PET scanner for each question. The “rating differences” were distributed roughly symmetrically. Normal distribution was not observed, which ruled out application of t-test. Probability of error was set to *p* < 0.05.

Comparison of the median scan experience ratings of the cohorts that underwent scanning exclusively on the SAFOV PET/CT scanner or exclusively on the LAFOV PET/CT scanner was performed by Mann-Whitney U test. Probability of error was set to *p* < 0.05.

For Wilcoxon signed rank tests and Mann-Whitney U tests two-tailed testing was applied if there was no reasonable thesis for one of the scanners “performing better” with respect to the specific items. One-tailed testing was applied for the items: “Was the duration of the scan bearable?”, “Are you convinced that the examinations performed with this scanner are state of the art?”, “Do you find the equipment in the examination rooms and scanner suitable for the most modern medical diagnostics?” and “Did you experience urinary urgency”. For these items the newer and faster (on average 15 min. scan time reduction) LAFOV scanner was expected to perform better than the old SAFOV scanner model.

## Results

### General study acceptance and feasibility

During the recruitment period, 2,001 adult patients who were clinically indicated for a PET/CT examination were eligible to participate in the questionnaire study. An additional inclusion criterion was having sufficient knowledge of German to complete the questionnaires. 52.3% (*n* = 1,047) agreed to participate in the study. Of these, 42 cases without documented consent and two cases with completely unanswered questionnaires were not included. This resulted in 1,003 participating patients with at least one usable questionnaire. The study sample did not differ from the total of all PET/CT subjects in terms of age, sex, and diagnoses (not shown). The response rate was not significantly affected by age or gender (not shown). As PET/CT scans were sometimes performed several times per patient, 1,115 questionnaires were available, of which 65% were completed in full. In the remaining 35% of the questionnaires, some questions were not answered, but all answered questions on the VAS sheets were included in the analysis. Unless otherwise stated, the first completed questionnaire was used for the analyses.

### How do patients experience the PET/CT scan and what side effects are most commonly reported?

When summarizing the subjective perception regarding the information received, the care experienced, anxiety, the duration of the examination and measurement, positioning and confidence in the equipment, their distribution on the VAS was unimodal with 68–82% (mean 76%) of ratings in the top decile. This indicates that the majority of patients coped well to very well with the PET/CT examination when considering the median of the VAS scores. However, about 9% of all patients gave negative to very negative ratings (score < 50%) in at least one question (Table 2A). The vast majority reported no relevant side effects (68%). However, the VAS data on the side effects experienced show skewed, rather bimodal distribution patterns. Claustrophobia, headache, pain, etc. were always experienced by a small number of patients during the scan. Most notable were the scores for tension and urinary urgency, where about 19% and 21% of the given scores fell into the rather severe discomfort range (score > 50) (Table 2B). In total, 32% of all patients experienced at least one negative side effect (score < 50).

**Table 2:**
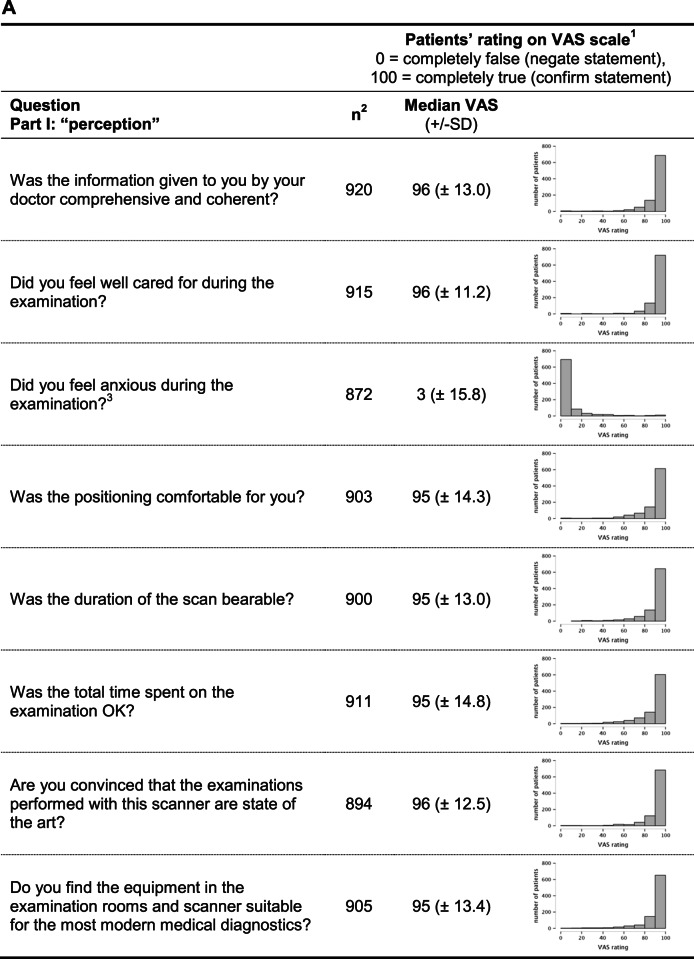

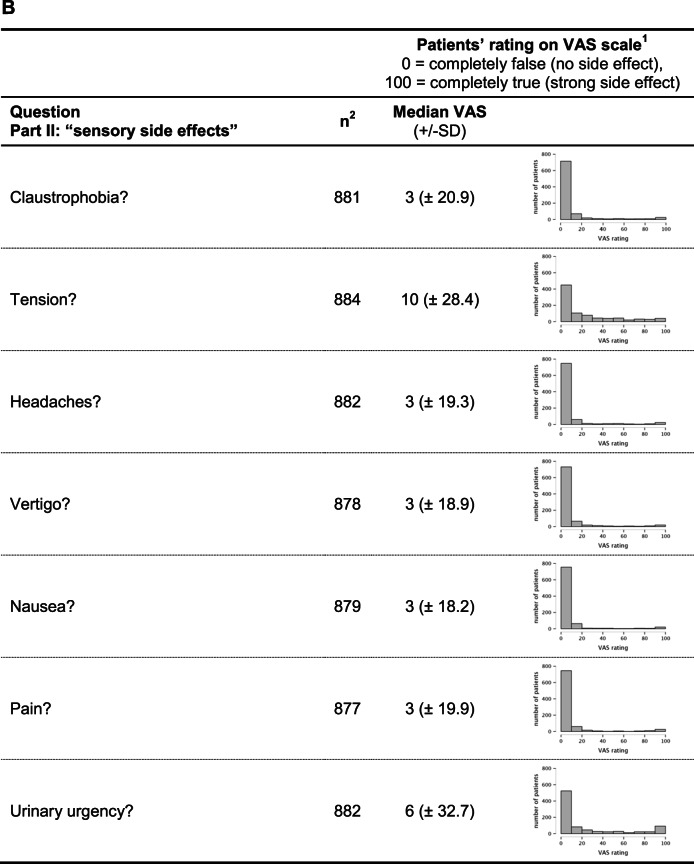
Self-reported PET/CT-related perceptions

^1^Patients' ratings of subjective perception (Table 2A) and side effects (Table 2B) for PET/CT examinations. The VASs were anchored by a negating descriptor (“completely false”) at 0, , and a confirming descriptor at 100 (“completely true”). ^2^Number of evaluable answers regarding the first recorded PET/CT scan during this study at both scanners (total cohort n=1,003). ^3^Original question on used questionnaire: “Were you free from anxiety during the examination?” This scaling has been reversed for the purposes of this evaluation to avoid double negation when discussing this item.

*Abbreviations*: *SD* standard derivation, *VAS* visual analog scale.

When looking at the context between psychological distress and the examination experience, the increased tendency towards depression only played a role in the evaluation of positioning and the feeling of “being well cared for”: an increase of one point on the 6-point depression scale resulted in an approximately 18% worse evaluation of the “positioning” (Table 3C) and 16% worse evaluation of “being well cared for” (Table 3B). If the patients were more likely to be burdened with worries, the perceived comprehensibility of the patient information was worse (Table 3A). However, the most striking and significant effect was that of the acute tension during the scan, as measured using the VAS questionnaire immediately after the examination (Table 3B-G): Increased tension during the scan was a negative predictor for all of the assessments queried, and this was most evident in the tolerability of the scan duration: an increase in tension of 10% points reduced this by 16% (Table 3D). In addition, claustrophobia plays a role in the feeling of “being well cared for” as well as in the assessment of device suitability (Table 3B und F).

**Table 3:** A-G. Possible impact factors on patient comfort

A Was the information given to you by your doctor comprehensive and coherent?			B Did you feel well cared for during the examination?		
Predictor	OR^1^	p^2^	Predictor	OR^1^	p^2^
Age	**0.88** ^3^	0.013*	Age	**0.82** ^3^	<0.001*
Sex (female)	1.10	0.544	Sex (female)	1.27	0.177
Depression	0.92^4^	0.276	Depression	**0.84** ^4^	0.041*
Anxiety	0.94^5^	0.495	Anxiety	0.98^5^	0.793
Worries	**0.90** ^6^	0.040*	Worries	0.93^6^	0.228
General Tension	0.95^7^	0.250	General Tension	1.06^7^	0.252
Tension (VAS)	-	-	Tension (VAS)	**0.90** ^8^	0.003*
Claustrophobia (VAS)	-	-	Claustrophobia (VAS)	**0.90** ^8^	0.031*
Urgency (VAS)	-	-	Urgency (VAS)	1.00^8^	0.921
C Was the positioning comfortable for you?			D Was the duration of the scan bearable?		
Predictor	OR^1^	p^2^	Predictor	OR^1^	p^2^
Age	0.93^3^	0.211	Age	0.90^3^	0.068
Sex (female)	1.40	0.059	Sex (female)	**1.64**	0.006*
Depression	**0.82** ^4^	0.027*	Depression	0.97^4^	0.763
Anxiety	1.02^5^	0.849	Anxiety	0.92^5^	0.400
Worries	1.01^6^	0.867	Worries	0.96^6^	0.547
General Tension	0.95^7^	0.364	General Tension	1.02^7^	0.767
Tension (VAS)	**0.88** ^8^	<0.001*	Tension (VAS)	**0.84** ^8^	<0.001*
Claustrophobia (VAS)	0.91^8^	0.078	Claustrophobia (VAS)	0.96^8^	0.433
Urgency (VAS)	1.00^8^	0.953	Urgency (VAS)	0.95^8^	0.077
E Was the total time spent on the examination OK?			F Are you convinced that the examinations performed with this scanner are state of the art?		
Predictor	OR^1^	p^2^	Predictor	OR^1^	p^2^
Age	0.95^3^	0.417	Age	**0.81** ^3^	<0.001*
Sex (female)	**1.60**	0.006*	Sex (female)	**1.49**	0.024*
Depression	0.91^4^	0.286	Depression	0.97^4^	0.697
Anxiety	0.97^5^	0.728	Anxiety	0.99^5^	0.878
Worries	1.00^6^	0.770	Worries	0.99^6^	0.116
General Tension	1.00^7^	0.755	General Tension	1.00^7^	0.623
Tension (VAS)	**0.90** ^8^	0.001*	Tension (VAS)	**0.93** ^8^	0.043*
Claustrophobia (VAS)	0.94^8^	0.206	Claustrophobia (VAS)	**0.90** ^8^	0.043*
Urgency (VAS)	1.01^8^	0.769	Urgency (VAS)	0.96^8^	0.137
G Do you find the equipment in the examination rooms and scanner suitable for most modern medical diagnostics?					
Predictor	OR^1^	p^2^			
Age	0.90^3^	0.059			
Sex (female)	**1.50**	0.019*			
Depression	0.90^4^	0.235			
Anxiety	0.95^5^	0.588			
Worries	1.00^6^	0.796			
General Tension	1.00^7^	0.698			
Tension (VAS)	**0.93** ^8^	0.031*			
Claustrophobia (VAS)	0.92^8^	0.088			
Urgency (VAS)	0.99^8^	0.681			

The interactions of various predictors on the score of the question asked (A-G) are shown as odds ratios. In addition to age and sex, other predictors were asked in standardized questionnaires about depression, anxiety, worry and general tension (here, patients were asked to consider the period of the last two weeks before the scan). The predictors tension, claustrophobia and urinary urgency, which were assessed by VAS, consider the acute sensations during the scan

A logistic regression model has been applied. ^ 1^OR: Odds Ratio, ^2^p: Probability of error in Wald Test, p<0.05 was considered significant (*). ^3^OR per 10-years increase in age, ^4^OR per 1-point increase in PHQ-2 Depression Scale (0-6), ^5^OR per 1-point increase in GAD-2 Anxiety Scale (0-6), ^6^OR per 10-point increase in PSQ-20-Worries Scale (0-100), ^7^OR per 10-point increase in PSQ-20-Tension Scale (0-100), ^8^OR per 10-point increase in VAS Scale (0-100)

### Do age or sex influence the reported experiences of the PET/CT scan?

In our study, the sex of the patient is one of the most important predictors of how the PET/CT examination is experienced. The odds for women to find both the duration of the scan and the total examination time more tolerable was 1.6-fold higher than for men (Table 3D and E). Patients’ age plays a role in their assessment of the comprehensibility of patient information and their feeling of being well cared for during the examination. Thus, an increase in age by 10 years is reflected in an 12% worse evaluation of the understandability of the patient informationand a 18% lower evaluation of being taken care of (Table 3A and B). Interestingly, older patients also tend to show a certain skepticism towards the scanner itself (Table 3F). In contrast, no correlation could be shown between positioning in the scanner and age (Table 3C-E).

### Does psychological distress play a role in the experience of the PET/CT examination?

**Fig. 1:**
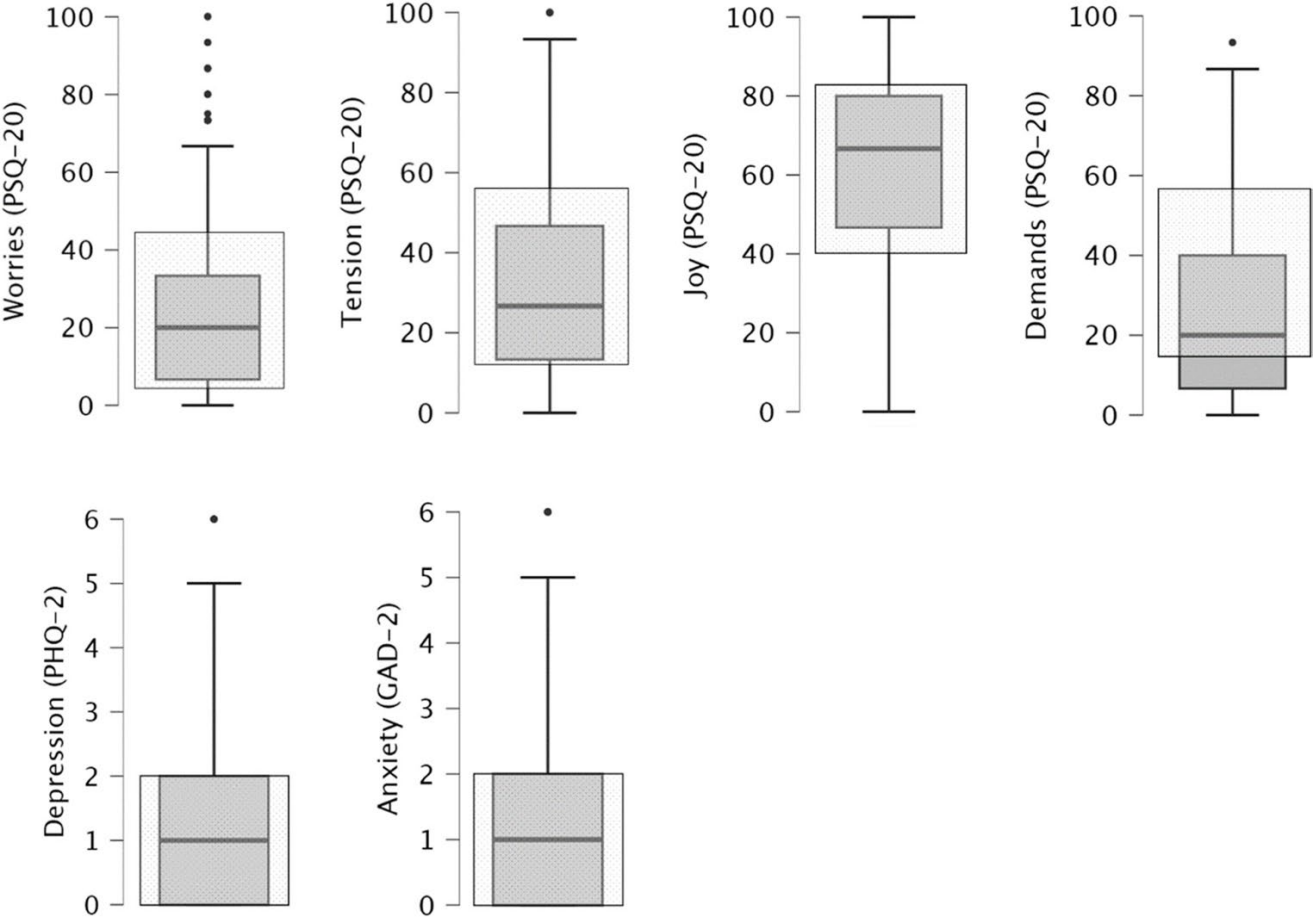
Self-reported general psychological condition of patients

In order to determine the general psychological state of the study patients, they were asked to complete appropriate standardized questionnaires before the PET/CT examination. Here, our study sample did not differ significantly in the information on worries, tension, joy, demands, depression and anxiety, from the information provided by healthy adults who were surveyed by Fliege et al. to validate these questionnaires (Fig. 1) [22].

Figure 1 The boxplots show patients’ ratings of their general psychological condition within the last 2–4 weeks before examination. The “outer” dotted boxes indicate the normal ranges of the respective scores for healthy adults [22]. PSQ-20 scores (top) range from 0 (e.g. no tension) to 100 (e.g. maximal tension). PHQ-2 and GAD-2 scores (bottom) range from 0 (e.g. no anxiety) to 6 (e.g. maximal anxiety).

Given that acute tension is so important to the overall experience of the examination, we wanted to see which predictors mattered here: Age and sex did not play a significant role as predictors for acute tension during the scan whereas higher values in the standardized scores for anxiety (OR:1.38; *p* < 0.001) and worries (OR:1.13; *p* < 0.039) predicted increased tension during the scan.

### Does disease severity influence the experience of the PET/CT examination?

To answer the question about the influence of disease severity, information on the largest group of patients studied, those with prostate cancer, was used. The Gleason score was used as a measure of disease severity. In prostate cancer patients (*n* = 243), only one significant correlation was found between a higher Gleason score and the associated perception of less comfortable positioning (OR: 0.77; *p* = 0.042). Just below the significance threshold, there was a possible association between a one-point increase in Gleason score and a 20% increased likelihood of answering the question “Did you feel well cared for during the examination?” in the negative. (OR: 0.79; *p* = 0.053). No other significant correlations were observed (data not shown).

### Are there differences between SAFOV and LAFOV scanners in terms of the comfort as reported by patients?

As a new LAFOV PET/CT was installed during the study period, it was possible to compare statements from patients who had been examined on both scanners. In this intra-individual comparison, the tolerance of scan time and the assessment of the equipment as adequate were rated as significantly better for the LAFOV PET/CT. An inter-individual comparison of patients measured only on the SAFOV scanner or only on the LAFOV scanner confirmed this difference. There were no other differences, e.g. with regard to positioning (Table 4, Part I). With regard to side effects, the LFOV PET was rated better for claustrophobia and urinary urgency in the intra-individual comparison, the latter was confirmed in the inter-cohort comparison (Table 4, Part II). Although the improvement appears small in percentage terms (approximately 2–12%), even minor improvements are significant, given that the approval ratings on the VAS scales are already very high at around 90%. Furthermore, the standard deviations in the intra-individual comparison demonstrate that patients benefited from the device change to varying degrees.

**Table 4:** Patient comfort depending on PET/CT scanner

	Patients with consecutive scans in SAFOV and LAFOV scanner^1^ Wilcoxon signed rank test			Comparison of cohorts scanned in SAFOV vs. cohort scanned in LAFOV scanner^2^ Mann-Whitney U test						
Question Part I “perception”	**N**	**P**^3^	**% improved rating LAFOV (±SD)**^4^	N_mCT_	N_Quadra_	**P**^3^	**mean VAS ratings (±SD)**	**% improved rating LAFOV**^5^		
Patient information comprehensive?^6^	77	0.197		503	336	0.250				
Feeling well cared for?^4^	77	0.582		503	333	0.716				
Anxious during the examination?^6^	65	0.644		473	324	0.611				
Patient positioning comfortable?^6^	72	0.317		489	335	0.120				
Scan duration well tolerated?^7^	68	0.004*	**4.4 (±11.1)**	489	333	0.044*	LAVOF 91.9 (±11) SAFOV 90.3 (±14)	**1.6**		
Total time spent on the examination OK?^6^	74	0.209		493	338	0.299				
Examinations performed with this scanner are state of the art?^7^	77	0.072		481	332	0.012*	LAVOF 93.1 (±11) SAFOV 91.2 (±14)	**1.9**		
Equipment, rooms and scanner suitable for modern diagnostics?^7^	76	0.010*	**2.9 (±18.1)**	488	338	<0.001*	LAVOF 92.6 (±12) SAFOV 89.9 (±14**)**	**3.7**		
Question Part II “sensory side effects”										
Claustrophobia?^6^	76	0.017*	**5.2 (±19.3)**	473	327	0.286				
Tension?^6^	76	0.246		477	327	0.723				
Headaches?^6^	79	0.826		476	326	0.820				
Vertigo?^6^	78	0.575		470	329	0.282				
Nausea?^6^	77	0.944		475	327	0.884				
Pain?^6^	75	0.322		474	326	0.872				
Urinary urgency?^7^	76	0.026*	**9.9 (±32.6)**	478	325	<0.001*	**LAVOF 16.6 (±26)** **SAFOV 29.0 (±36)**	**12.4**		

^1^Intra-individual comparison: All patients underwent both a SAFOV scan and a subsequent LAFOV scan. The questionnaires for the last SAFOV PET examination and the next LAFOV PET examination were compared. 'N' indicates the number of cases in which the corresponding question was answered by the same patient for both measurements. ^2^Inter-individual comparison: questionnaires from two cohorts were compared, one scanned with the SAFOV PET and the other scanned with the LAFOV PET. Here, 'N_mCT_' and 'N_Quadra_' refer to the number of cases in which the respective question was answered by a patient who underwent examination on the corresponding scanner.^3^Probability of error in Wilcoxon signed rank test (left) and Mann-Whitney U test (right). ^4^Mean and SD of the individual rating difference in % between LAFOV and SAFOV. ^5^Difference between the mean ratings on VAS scales of the LAFOV and the SAFOV groups (sub-cohorts).^6^Two-tailed test.^5^One-tailed test: here, the LAFOV scanner was expected to perform better than the SAFOV scanner. *p<0.05: Comfort in the LAFOV scanner rated higher than in the SAFOV scanner

## Discussion

The main aim of this questionnaire-based study was to ask over 1,000 patients about their PET/CT scan experience and any side effects. Fortunately, most patients tolerated the scan well without relevant side effects. However, around 9% reported an unpleasant experience, equating to 9–10 patients per week at our PET/CT centre. The survey aimed to identify these patients and explore ways to improve their experience. Self-reported stress and depression, as well as demographic data were used to determine predictors of poor examination experiences, with the goal of pre-identifying patients who may need more support. The fact that the new LAFOV scanner was installed at our PET/CT centre as a successor to the SAFOV scanner during the study enabled a comparison of the two devices from the patient’s perspective.

Although many studies show that cancer patients are more likely to experience depression, particularly women and those under 50, our patient group showed no significant differences compared to healthy individuals in terms of depression, anxiety, or worry [8, 14]. This may be partly due to the high proportion of prostate cancer and melanoma patients in our sample (43%), as these groups generally report lower depressive symptoms [27, 28]. Additionally, as the median time since diagnosis was 22 months, patients may have adjusted to their condition. Consequently, anxiety did not predict a poor scan experience.

A review of ten studies on anxiety during PET/CT scans revealed that patients experience high levels of anxiety, but there are no consistent links to gender, demographics, or disease stage [1]. Educational level, internet research, and whether it was a first or repeated scan also had only little influence [10]. However, many of these studies had small sample sizes and used non-standardized tools. In contrast to anxiety, our study found that higher levels of depression symtomes and older age clearly predicted a general sense of not being well cared for. Since anxiety states are acute and often become apparent at short notice, while depressive experiences tend to be less obvious, it makes sense to examine both as predictors of difficulties for patients undergoing PET/CT examinations. As the PHQ-4 score is a quick and reliable indicator of depression [21], it may be useful to administer it prior to the examination in order to identify critical patients in need of additional support.

Contrary to our expectations, age did not significantly impact on scan positioning or endurance. Older patients did, however, have more difficulty understanding the information provided and tended to be more sceptical of new technology - issues that could probably be addressed by devoting more time to patient information. Men were significantly less tolerant of long scan duration than women, a finding consistent with previous studies [1, 8]. Women, influenced by social expectations, may be more likely to give positive responses, a phenomenon known as social desirability bias, which could have affected the results of our study [29–31].

It is not surprising that the LAFOV scanner, as a new purchase, was considered to be more state of the art. However, it is more important to note that reducing scan time is crucial for improving patient comfort. Smaller studies comparing PET/CT and PET/MRI investigations with 30–120 participants identified positioning and scan time as the main causes of discomfort [8, 12, 32, 33]. Our device comparison strongly supports this: examination time with the newer LAFOV PET/CT was, on average, 15 min shorter than with the SAFOV PET/CT [19, 34, 35], and scan time was actually better tolerated in both intra- and inter-individual comparisons. The markedly increased sensitivity of LAFOV PET scanners provides further opportunities for protocol optimization. Although the injected activity and acquisition time were already substantially reduced in this study compared to conventional SAFOV protocols, we intentionally maintained a balanced approach between patient dose and scan duration. Prior work has demonstrated that substantial dose reductions (e.g. from 3 MBq/kg to 1 MBq/kg for [^18^F]FDG) can provide comparable diagnostic accuracy to standard protocols [19, 34, 35]. Conversely, in scenarios where examination time is the primary constraint, maintaining a standard activity while further shortening the acquisition time could be a feasible alternative, whilst ensuring that the radiation exposure does not exceed that typically associated with SAFOV systems.

It is likely that the LAFOV scanner’s superior performance in terms of urgency and claustrophobia is also due to the shorter scan times. Side effects such as nausea and headaches, which might be less sensitive to scan time, were not rated differently in the device comparison. This also included acute tension which was the strongest predictor of an unpleasant PET/CT experience.

High tension correlated with poorer perceptions of care, discomfort in positioning, poorer scan time tolerance, and less trust in the technology. Contributing factors may include a lack of procedural knowledge, radiation concerns, or anxiety about results. Providing more detailed pre-scan information could help in this regard. Other comfort measures that have been suggested include vacuum positioning devices, although these may increase radiation exposure for staff [36], and relaxation tools such as music or videos. Informational content during tracer uptake could also ease patient concerns [11]. Effective communication between technologists and patients, including during the scan, is essential for reducing stress [37]. More intensive care for children requiring cross-sectional imaging is already widely implemented in paediatric radiology. Intensive coaching is provided for caregivers and children prior to the examination if necessary, and this sometimes involves the use of animations, games, films and model scanners. This is also considered an effective method of reducing the need for anaesthesia [38]. Providing appropriately adapted coaching for adults as a ‘service’ when needed would certainly be desirable, albeit personnel- and time-intensive. A feasible possibility would be to provide specialised apps or movies to help prepare for PET/CT examinations.

The study has several limitations. Selection bias is a possibility, as non-participants may differ from participants in psychological terms or in terms of their scan experiences. Social desirability bias may also have led to overly positive evaluations. We lack data on whether it was a patient’s first scan, although repeated scans did not show differences in scores. Some study participants will inevitably not want to or be able to answer all the questions in the questionnaire recording anxiety, depression and stress. Although imputation is a common procedure for partially incomplete questionnaires, it is not without potential error. Furthermore, disease severity was only investigated in the prostate cancer patient group, as it was the largest and most homogeneous group, with the most standardised staging. It was not possible to conduct a comparable analysis for women because they were too heterogeneous in terms of their diagnoses. The study spanned 19 months, during which time adjustments to the protocol (e.g., lower doses of contrast media and mannitol) may have impacted patient experience by reducing urinary urgency, for example.

In summary, general psychological stress, worry, and anxiety had less of an impact on PET/CT scan experiences than expected. Acute tension during the scan had a larger effect, particularly on positioning and tolerance of scan time, and also influenced perceptions of the technology. Clinically significant depression and older age were associated with feeling less well cared for. Men showed less tolerance of scan duration, though age had limited influence.

In conclusion, it can be stated that a non-negligible proportion of patients experience greater stress from the PET/CT examination than others. This group includes older male patients and those prone to depression. Particular attention should be given to this group when information is provided before and during the examination. It would also be useful to test and establish measures to reduce tension for all patients undergoing the examination. Reducing in scanning time, which is already possible with LAFOV scanners, is undoubtedly a key factor in improving patient comfort.

## Data Availability

The data presented in this study are available on request from the corresponding author. The data are not publicly available due to privacy and ethical reasons.
